# Performance Evaluation of Community Hospitals in Thailand: An Analysis Based on the Balanced Scorecard Concept

**Published:** 2020-05

**Authors:** Somnuk AUJIRAPONGPAN, Kanookwan MEESOOK, Pornpan THEINSATHID, Chanidapa MANEECHOT

**Affiliations:** School of Management, Walailak University, Nakorn Sri Thammarat, Thailand

**Keywords:** Performance, Balanced scorecard, Community hospitals

## Abstract

**Background:**

Although the concept of the Balanced Scorecard (BSC) was designed for profit-based organisations, the application of the BSC in public and nonprofit organisations (NPOs) could be performed within the NPOs and public health sector, as the conceptual foundation of this system was developed from community hospitals performance.

**Methods:**

This study used the BSC concept to analyse the 16 key performance indicators and trends of performance during the last five years of 52 community hospitals located in upper southern Thailand in 2017 and tendency of 2013–2017. The instruments included an annual report and a questionnaire. A statistical analysis to determine percentages, means and standard deviations was performed.

**Results:**

The major findings of the community hospitals performance were as follows: Customer perspective: 1. Patient complaint rate of 0.0097% and 2. Outpatient waiting time of 91.89 minutes, Financial perspective: 1. Ratio of total revenue to total expense at 0.9949 and 2. Cost of drugs and materials to total expense at 13.32%, Internal process perspective: 1. Bed turnover at 88.16 and 2. Hospital infection rate of 0.379 times:1,000 patient days, Learning and growth perspective: 1. Staff turnover rate of 4.69% and 2. Number of research studies at 3.77 articles. Trends and performance of hospitals in every perspective of the BSC in the last 5 years showed no differences.

**Conclusion:**

The community hospitals offer services such as treatment of common diseases. If the community hospitals could not assist, they will go to referral system by referring patients to secondary and tertiary care respectively.

## Introduction

Community Hospitals (CHs) are organisations for healthcare services operated by the government. They comprise district hospitals in 780 locations all over Thailand (1). They have the smallest size among hospitals under the Ministry of Public Health and are health units providing medical and public health services at the district level with approximately 10–150 beds for patients and regular physicians as well as other public health staff. The services emphasise diagnosis, medical treatment and rehabilitation as well as health promotion services, sanitary services and disease prevention. These hospitals also provide patient referral services for further treatment while gathering statistics and data for presentation to the provincial public health sector.

According to the study of Thai community, hospitals assessed performance is based on HA standards. However, they were confronted with work effectiveness and health services problems, such as manpower, service facilities, medication, technology, and finance as well as problems stemming from the outcomes of the health service system, effectiveness and the quality of service and fairness of the health service system. To resolve the current problems, adapt to present circumstances and be competitive among health service systems with more efficient improvements, it is necessary for community hospitals to have alternative performance measurement tools with more effectiveness to accommodate performance management. The tool that is most popularly used is the BSC, which was invented by Kaplan and Norton in 1992 (2). It was a new invention to measure the efficiency of work, and an instrument for performance management. The BSC comprises two dimensions: financial and non-financial dimensions. These dimensions consist of four perspectives: financial perspective, customer perspective, internal process perspective and learning and growth perspective. Although it was designed for profit-based organisations, the application of the BSC in public and nonprofit organisations could be performed within the NPOs and government sectors, as the conceptual foundation of this system was developed from performance assessment and strategic management, which are universal trends of current work management (2, 3).

This tool originated from the concept of helping to communicate strategies for organisations with practical implementation. The measurements of practices were divided into two dimensions, including financial measurement and non-financial measurement consisting of two perspectives: financial perspective, customer perspective, internal process perspective, and learning and growth perspective (2).

With these concepts, the conceptual foundation of BSC was developed for evaluation of community hospitals performance.

## Materials and Methods

The four most important strategic dimensions on the card for health care system were organizational health, quality and process improvement, volume and market share growth and financial health. Kaplan stated in Strategic Performance Measurement for Nonprofit Organisations in 2001 that the missions and visions of government organisations and non-profit organisations are different from non-governmental organisations. Therefore, it is difficult to apply an original balanced scorecard perspective with a financial perspective at the top, as financial success is not the main objective of such organisations. Thus, it is necessary to improve it by selecting customer perspective as the top (3). In fact, nonprofit organisations should consider determining the missions and strategies at the top of their BSC to cover all indicators from the aspects of the BSC (4). This would reflect the long-term goals of the organisations that they could improve through indicators to make the goal more achievable (5).

From the literature review, the concepts, theories and relevant studies included applications of the balanced scorecard for strategic management and performance measurement in the health sector (6). Configuring the balanced scorecards was carried out for measuring health system performance: Hospital Management and the Balanced Scorecard for healthcare in China and Japan (7), Evidence from a 5-year evaluation in Afghanistan (8), Design of a balanced scorecard on non-profit organisations (9–11), Implementing a balanced scorecard in a not-for-profit organisation (5, 11), Performance measurement in the hospitals by the balanced scorecard (5,13), Selecting hospital key performance indicators using the Analytic Hierarchy Process Technique (14), Identifying key performance indicators for holistic hospital management with a Modified DEMATEL Approach (15), Key performance indicators in hospital based on balanced scorecard model (16, 17) and Application of the balanced scorecard for an academic medical centre in Taiwan: The effect of warning systems on improvement of hospital performance (18). The researcher could synthesise 16 key performance indicators (KPI) for hospitals covering the BSC in two dimensions, including financial and non-financial measurements, consisting of two perspectives: the financial perspective, customer perspective, internal process perspective and learning and growth perspective (19). These were used to assess the performance of community hospitals in the upper southern region of the country, as shown in Table 1.

**Table 1:** Structure of hospital key performance indicators on the BSC

***Perspective***		***KPI***
Customer	C1	Rate of patient complaints
	C2	Patient satisfaction percentage
	C2-1	Inpatient satisfaction percentage
	C2-2	Outpatient satisfaction percentage
	C3	Outpatient waiting time
Finance	F1	Ratio of total revenue to total costs
	F2	% Personal costs of total costs/Total cost
	F3	% Cost of drugs and materials/Total cost
	F4	% Training costs to total costs/Total cost
Internal Process	P1	Average length of stay
	P2	Bed turnover
	P3	Bed occupancy
	P4	Hospital infection rate
	P5	Mortality rate
	P6	Readmission rate
Learning and Growth	L1	Staff satisfaction rate
	L2	Staff turnover
	L3	Number of studies

The research is utilised to examine the performance of community hospitals in the upper southern region of Thailand in 2017 and tendency of 2013–2017. The analysis is based on the balanced scorecard concept. The conceptual framework was processed from the concept of the Balanced Scorecard (BSC) (2). Populations in this research comprised 52 community hospitals in the upper southern region of Thailand according to classification criteria from the Public Health Management Division, Office of the Permanent Secretary of Ministry of Public Health. The instruments included an annual report of 2013–2017 and a questionnaire for data correction from directors of 52 community hospitals. The statistics in this study were descriptive statistics, including frequencies, percentages, means and standard deviations for analysing the performance and tendency of community hospitals for the 16 KPIs. It covered 4 perspectives of the BSC, including the customer perspective with 3 indicators: patient complaint rate (C1), satisfaction percentage rate (inpatient/outpatient) (C2), and outpatient waiting time (C3) as well as the financial perspective with 4 indicators: ratio of total revenue to total expense (F1), percentage personal expense of total expense (F2), percentage cost of drugs and materials to total expense (F3), and percentage training expense to total expenses (F4). The internal process perspective consisted of 6 indicators: average length of stay (P1), bed turnover (P2), bed occupancy rate (P3), hospital infection rate (P4), mortality rate (P5) and readmission rate (P6), and finally, the learning and growth perspective with 4 indicators: staff satisfaction rate (L1), staff turnover rate (L2), and number of researchers (L3).

## Results

Performance measurement is common in healthcare. Balanced scorecards are used in health care to list the results of the delivery of health care services as a continuous quality improvement approach. The following section presents the findings from the case study.

### Performance of community hospitals in 2017 Customer perspective

The mean patient complaint rate was 0.0097% or 9.7:1,000 persons. The mean patient satisfaction rate was 86.75%, and the mean outpatient satisfaction rate was 83.57%.

Outpatient waiting time refers to the time patients waited, which covered the duration from card making to being seen by a doctor or having outpatient treatment. The mean outpatient waiting time was 91.89 minutes. The shortest duration was 20.03 minutes, while the longest duration was 261.00 minutes.

### Financial perspective

The ratio of total revenue to total expenses was 0.9949, meaning that, in general the hospitals faced loss issues. When considered by hospital, 31 hospitals (60.78%) had profit, while 20 hospitals accounted for 39.22% of loss turnover. The percentage of personal expense to total expense was 40.11% of total expense. The percentage of cost of drugs and materials to total expense was 13.32% of total expense. The percentage of training expense to total expense was 0.58% of total expense.

### Internal process perspective

The average length of stay was 3.281 days, with a minimum of 2.000 days and a maximum of 10.730 days. The mean of the bed turnover rate was 88.168, with proper bed usage. However, when considered by hospitals, more than half of all community hospitals (51.92%) had unworthy bed turnover rates and needed to adjust their service system (bed turnover < 80). The mean of the bed occupancy rate was 77.824, with unworthy bed usage (bed occupancy < 80) implying service systems should be improved. The mean hospital infection rate was 0.379 times:1,000 patient days, with a minimum of 0.000 times:1,000 days and a maximum of 1.020 times:1,000 days. The mean mortality rate was 2.298 people, with a minimum of 2 people and a maximum of 391 people. The mean readmission rate over 28 days was 6.307%.

### Learning and Growth Perspective

The staff satisfaction rate for staff working in the hospitals averaged 70.20%. The mean staff turnover rate was 4.69. The mean number of studies was 3.77 titles or articles in 1 year, with a minimum of 0 articles and a maximum of 18 articles.

### Tendency of 5-year performance in 2013–2017 Customer Perspective

The mean 5-year patient complaint rate was 0.0121% or 12.1:1,000 patients, with a total change of −0.0919. The mean 5-year inpatient satisfaction rate was 86.24%, with no difference in change of +0.0007%. The mean 5-year outpatient satisfaction rate was 83.95%, without significant change of −0.0002%. The mean 5-year outpatient waiting time was 93.60 minutes with a change of −0.0657% from the graph, while the mean outpatient waiting time was the lowest at 79.83 minutes (Table 2).

**Table 2:** Performance and tendency of 5-year in 52 community hospitals

***BSC-KPIs***		***2013***	***2014***	***2015***	***2016***	***2017***	***Mean***	***%Change***
		Customer Perspective						
C1	Person	0.012	0 017	0.011	0 . 010	0.010	0.0121	− 0.0919
C2-1	%	86.494	85.734	86.009	86.199	86.754	86.2379	+ 0.0007
C2-2	%	83.597	83.697	85.201	83.678	83.569	83.9481	− 0.0002
C3	%	110.314	79.834	96.874	89.095	91.888	93.6010	− 0.0657
		Financial Perspective						
F1	Ratio	1.003	0.972	0.991	1.028	0.995	0.998	− 0.0024
F2	%	49.251	52.636	46.802	47.770	40.110	47.314	− 0.0578
F3	%	13.682	11.642	15.178	13.356	13.321	13.436	− 0.0203
F4	%	1.681	1.294	0.995	0.959	0.576	1.101	− 0.3253
		Internal Process Perspective						
P1	Day	2.964	2.990	2.947	3.238	3.281	3.084	+ 0.0243
P2	%	91.155	86.876	85.639	94.094	88.168	89.186	− 0.0103
P3	%	79.131	75.335	75.757	83.671	77.824	78.344	− 0.0063
P4	times:1,000 patient days	0.601	0.353	1.315	0.777	0.379	0.685	− 0.4280
P5	%	39.100	16.029	25.750	24.545	27.298	26.544	− 0.2525
P6	%	6.042	5.277	6.058	5.016	6.307	5.740	− 0.0048
		Learning and Growth Perspective						
L1	%	76.341	77.558	77.259	73.407	70.202	74.954	−0.0216
L2	%	7.475	4.460	4.712	9.108	4.691	6.089	−0.2703
L3	No. Studies	4.958	4.679	2.758	2.871	3.767	3.806	−0.1198

### Financial Perspective

The mean of the 5-year ratio for total revenue to total expense (F1) was 0.998 persons, with a change of −0.0024%. This means that, on average, the community hospitals were confronted with loss issues. When considered by years, the community hospitals had profits in 2013 and 2016 only. The mean 5-year percentage personal expense of total expense was 47.314%, with a change of −0.0203%. The mean 5-year percentage cost of drugs and materials to total expense was 13.436%, with a change of −0.0203%. The mean 5-year percentage training expense to total expense was 1.101, with a change of −0.3253%.

### Internal Process Perspective

The mean 5-year average length of stay was 3.084 days, with a change of +0.0243%.

The mean 5-year bed turnover rate was 89.186%, while total change was −0.0103% in the past 5 years; the community hospitals used beds properly. The mean 5-year bed occupancy rate was 78.344%, while total change was −0.0063%. In the past 5 years, the community hospitals utilized beds inefficiently and the service system should be adjusted. The mean 5-year hospital infection rate was 0.685 times: 1,000 days, with a change of −0.4280%. The mean mortality rate was 26.544 patients, with a change of −0.2525%. The mean readmission rate over 28 days was 5.740%, with a change of −0.0048%.

### Learning and Growth Perspective

The mean 5-year staff satisfaction rate was 74.954%, with a change of −0.0216%. When considered by year, the values were similar. The mean 5-year staff turnover rate was 6.089%, with a change of −0.2703%. When considered, the highest value was in 2016, when the staff turnover rate was 9.108%. The mean 5-year number of studies was 3.806 titles or articles, with a change of −0.1198%.

## Discussion

### Customer perspective

The patient complaint rate was extremely low, while the patient satisfaction rate was higher than 80% because of many causes. Such causes included that most community hospitals were secondary hospitals providing basic rather than complicated treatment. Most patients used 30 Baht universal health cards and were not wealthy. Thus, they did not expect a high level of service for treatment compared with large-scale hospitals or private hospitals. The patients just desired to be cured from the symptoms or disorders they had. If they were not made better, they would be referred to hospitals with more comprehensive and inclusive treatment. Additionally, channels for patient complaints were scarce. That is, the complaint channels were mostly complaint forms in the hospitals, complaints through webpages of the hospitals or other technology were barely available, making the results of complaints or satisfaction rates at a good level to meet the standards.

Outpatient waiting time was found on average to be 1.30 hours, which was relatively long compared with treatment at private hospitals. This was because the community hospitals had larger numbers of patients visiting to get treatment, while physicians and medical personnel tended to be in short supply in the upcountry or faraway areas. Mostly, only 1–2 physicians were on shift at community hospitals to provide treatment, compelling the patients to wait for a long time before examination.

### Financial perspective

On average, the hospitals had loss issues accounting for 60.78% of all community hospitals. This was because the community hospitals were nonprofit organisations. In addition, the government has a policy of universal health care, meaning every Thai national can access medical services thoroughly, including alien laborers working in Thailand. The Ministry of Public Health allocated a budget individually for both outpatient and inpatient care. The government asked people to register with the hospitals, so it could allocate budgeting for the hospitals by the number of patients and by rights. Charges from patients could not be accurately collected from the National Health Security Office (NHSO) in Thailand with capitation or whole payment for outpatient expenses. Therefore, if a patient receives many treatments, no matter how serious his/her condition or how expensive, it is still the sole responsibility of the hospitals. In the meantime, inpatients were in a disease-related group (DRG). DRG usage in payment for inpatients of the NHSO was that the hospitals reported disease groups and whole payment was made by such groups. For example, Caesarean section costs 4,000 Baht, which could be adjusted by symptoms called the adjusted relative weight or adjusted RW. Capitation for outpatients and DRG for inpatients allowed the NHSO not to accept any risks. It could evade risks with all health insurances to service providers. In fact, hospitals were coerced to provide services, as they could not refuse patients.

Accounting loss means lower revenue than expense. The public hospitals, especially the Ministry of Public Health, mostly had revenue from the NHSO or universal health care. However, the hospitals collected only 50–60% of the charges when providing services, while the costs were high, at approximately 70–80% of the service charges. Nevertheless, the public hospitals had loss or constant expenses that they could not avoid with a relatively high proportion, including personnel expenses (at more than half of all expenses), medicine and medical supply expenses, and expenses for building construction and medical equipment purchases.

### Internal process perspective

Bed occupancy rate indicates the effectiveness of bed usage as well as the efficiency of treatment service. It was obvious that first-level hospitals and first-level hospitals did not use beds efficiently. This was because they did not usually admit patients or there were few inpatients. Most of these hospitals provided treatment for general diseases without complexity or much severity. They had only general doctors and lacked specialists. When patients had severe illness or symptoms, the hospitals would refer them to other hospitals that were more prepared. As a result, their beds were not fully utilised.

### Learning and Growth Perspective

The staff satisfaction rate was high, while the staff turnover rate was low, as most staff were bureaucrats and regular employees with certain remunerations and welfare as well as stability in life. They were praised in society inside and outside the organisations. Their relationships with colleagues were good, since they received assistance from them. As a result, the staff satisfaction rate was high, while the staff turnover rate was low. This is consistent with the study of, who studied the satisfaction rate of staff at Khukhan Hospital, Khukhan District, Si Saket Province and found that the factors most affecting staff satisfaction included salary and welfare, work stability, advancement and promotion—all of which led to a low turnover rate (20).

## Conclusion

The community hospitals, it can be difficult to boost quality of care. Larger hospitals and health systems have more resources at their disposal, and community hospitals may lag behind. But that does not mean it is impossible for smaller hospitals to make progress – they just have to use different strategies to achieve success. From the research, there is no significant improvement for all dimensions during the period of 2013–2017. The management of community hospital should promote a high performance healthcare system as well as to improve health care practice and policy through discussion of the finding of the BSC at the community hospitals. The community hospitals are nonprofit organization should focus on how to improve patient satisfaction, waiting time, staff satisfaction rate and how to manage cost saving for further improvement by promoting a high performance healthcare system as well as to improve health care practice and policy through discussion of the finding of the BSC at the community hospitals.

## Ethical considerations

Ethical issues (Including plagiarism, Informed Consent, misconduct, data fabrication and/or falsification, double publication and/or submission, redundancy, etc.) have been completely observed by the authors.
